# Loss of Planar Cell Polarity Effector Fuzzy Causes Renal Hypoplasia by Disrupting Several Signaling Pathways

**DOI:** 10.3390/jdb10010001

**Published:** 2021-12-23

**Authors:** Irene-Yanran Wang, Chen-Fang Chung, Sima Babayeva, Tamara Sogomonian, Elena Torban

**Affiliations:** 1 Department of Medicine, McGill University, Montreal, QC H4A 3J1, Canada; yrwang25@hotmail.com (I.-Y.W.); aikenchung.bme@gmail.com (C.-F.C.); simabab@gmail.com (S.B.); tamara.sogomonian@mail.mcgill.ca (T.S.); 2 McGill University Health Center Research Institute, Montreal, QC H4A 3J1, Canada

**Keywords:** PCP effectors, kidney development, transcriptional profile, kidney anomalies

## Abstract

In vertebrates, the planar cell polarity (PCP) pathway regulates tissue morphogenesis during organogenesis, including the kidney. Mutations in human PCP effector proteins have been associated with severe syndromic ciliopathies. Importantly, renal hypoplasia has been reported in some patients. However, the developmental disturbance that causes renal hypoplasia is unknown. Here, we describe the early onset of profound renal hypoplasia in mice homozygous for null mutation of the PCP effector gene, *Fuzzy*. We found that this phenotype is caused by defective branching morphogenesis of the ureteric bud (UB) in the absence of defects in nephron progenitor specification or in early steps of nephrogenesis. By using various experimental approaches, we show that the loss of Fuzzy affects multiple signaling pathways. Specifically, we found mild involvement of GDNF/c-Ret pathway that drives UB branching. We noted the deficient expression of molecules belonging to the Bmp, Fgf and Shh pathways. Analysis of the primary cilia in the UB structures revealed a significant decrease in ciliary length. We conclude that renal hypoplasia in the mouse *Fuzzy* mutants is caused by defective UB branching associated with dysregulation of ciliary and non-ciliary signaling pathways. Our work suggests a PCP effector-dependent pathogenetic mechanism that contributes to renal hypoplasia in mice and humans.

## 1. Introduction

Planar cell polarity refers to the uniform cell arrangement along a tissue plane [1]. Originally discovered in *Drosophila melanogaster*, PCP genes control cellular cytoskeleton rearrangements, enabling generation of uniform arrays of cellular protrusions or of coordinated cell movements over long distances, which is critical for tissue morphogenesis (reviewed in [2]). Based on their specific roles in PCP establishment in *Drosophila*, the PCP genes have been classified into three major groups: the global, the core and the PCP effectors. The most numerous are the PCP effectors; expression of effectors differs in various cells, and they control a wide range of cellular processes. The four PCP effectors that regulate actin-based hair-like protrusions (trichomes) on fly wing cells are *Fuzzy* [3,4], *Inturned* [5,6,7], *Fritz* [7] and *multiple wing hairs* [8,9]. Localized at the proximal side of each wing cell, these PCP effectors interact with each other and restrict actin polymerization at the proximal side, permitting generation of a single trichome only at the opposite, distal, aspect of each wing cell [6,8,10,11].

PCP genes belonging to all three groups have been identified in vertebrates where they regulate numerous processes such as neural tube closure or heart outflow tract [2,12,13]. In preliminary experiments, we found that the homozygous inactivation of *Fuzzy* in mice causes profound kidney hypoplasia. Although mild renal dysplasia has been linked to the disturbance of global and core PCP genes [14,15,16,17,18], kidney malformations in the PCP effector gene mutants have not been investigated. Furthermore, the mechanisms by which PCP effector gene mutations might disturb renal development have not been explored.

One plausible mechanism involves an effect of PCP effectors on the primary cilium of renal cells. Loss-of-function mutations in *Fuzzy* or *Inturned* genes in mice lead to complex defects such as looptail, cardiac outflow defects and a spectrum of malformations commonly attributed to the dysfunction of primary cilia, such as cranial NTDs and polydactyly [19,20]. Indeed, the depletion of *Fuzzy* and *Inturned* was associated with abnormal ciliogenesis in *Xenopus* [21] and in respective mutant mouse tissues [19,20,22,23]. PCP effectors appear to regulate vesicle trafficking of certain cargo proteins required for ciliogenesis [24,25,26]. Hedgehog signal transduction, linked to the primary cilium, is dysregulated in *Xenopus Fuzzy* morphants [21] and in the spinal cord and developing limbs of mutant *Fuzzy* mice [20].

The development of the mammalian kidney is complex, and it is possible that PCP effectors might be involved in a wide array of critical developmental programs. The final metanephric kidney is initiated at ~E10.5 in mice or on the 35 day of gestation in humans. Signals secreted from cells of metanephric mesenchyme (MM) induce the ureteric bud (UB) to grow out from the caudal nephric duct and invade the MM [27]. Interactions between the UB and MM lineages induce repetitive branching of the UB that will generate the entire renal collecting system. Concomitantly, signaling molecules secreted by the UB cells cause the MM to condense around the UB tips and form a layer of Six2+ nephron progenitor cells (NPCs) [28] that give rise to early stages of the nephron: renal vesicles, comma- and S-shaped bodies [29], forming glomerulus and all tubular segments as differentiation proceeds. A complex system of transcription factors regulates expression of the growth factor signaling pathways that govern all stages of kidney development [29].

Branching morphogenesis depends on reciprocal UB-MM interactions. Glial cell-derived neurotrophic factor (GDNF) is secreted by the undifferentiated MM and binds to the tyrosine kinase receptor c-Ret, expressed by the cells of the nephric duct. GDNF/c-Ret interactions are critical for initial UB induction and continue to drive UB branching cycles throughout development [30,31]. Since each nephron forms at the UB tip, the number of branching events defines the final nephron number and affects overall kidney size and function [32,33]. Shh, Wnt/β-catenin and FGF signaling pathways have all been implicated in UB branching [34]. Self-renewal and differentiation of NPCs rely on the expression of several transcription factors, including WT1 and Six2 [28,35]. Disruption of either molecule leads to premature loss of NPCs and renal hypoplasia and/or renal agenesis [28,36]. However, a broad spectrum of other developmental programs must be considered to assess how mutant PCP effector genes might lead to congenital kidney anomalies in humans.

In this manuscript, we describe the early onset of profound renal hypoplasia in homozygous *Fuzzy* mutant mice. We show that renal hypoplasia is caused by deficient ureteric branching morphogenesis in the absence of detectable defects in nephron progenitor cell specification or in early stages of nephrogenesis. By combining unbiased RNAseq, bioinformatics, in situ hybridization and immunofluorescence, we demonstrate that the loss of *Fuzzy* affects several signaling pathways including Gdnf/c-Ret and Shh. In summary, we have identified a set of PCP effector pathways that are required for kidney development in mammals.

## 2. Materials and Methods

### 2.1. Mouse Breeding and Genotyping

Generation of the *Fuzzy* gene-trap mouse was previously described [22]. The insertion of a gene-trap β-Geo cassette into the third intron of the *Fuzzy* gene leads to the loss of the normal transcript. We consider this mutation a loss-of-function (null) mutation [20,22], and refer to it as *Fuzzy−/−*. Homozygous *Fuzzy−/−* mice were obtained by brother-sister mating of heterozygous littermates. Appearance of the plug is considered 0.5 days *post coitum*, dpc. Embryonic day 14.5 (E14.5) is the latest stage with the reliable survival of homozygous *Fuzzy−/−* embryos on the C57Bl6 background. The mice were housed at the McGill Transgenic Facility. Animal manipulations conform to the Canadian Animal Act. Animal protocol #7606-2015 was approved by the McGill University Animal Care Committee.

The DNA was extracted from tail biopsies of weaned mice or from embryo limb biopsies by using M-Fast Genotyping Kit (Zmtech Scientifique, Montreal, QC, Canada). The wildtype allele was amplified with forward primer “mFuzzy-Exon3-F” 5’-CACCTCTGAGCTGAGGCTGG-3’ and reverse primer “mFuzzy-Exon4-R” 5’-CTCAATTCTTTCTTCAGTCTTTC-3’, resulting in a 500 nucleotide PCR product. The gene-trap allele was detected by forward primer “βGeo-primer1-F” 5’-TTATCGATGAGCGTGGTGGTTATGC-3’ and reverse primer “βGeo-primer2-R” 5’-GCGCGTACATCGGGCAAATAATATC-3’, producing a 680 bp fragment. The PCR reaction was conducted in a PTC-100 Peltier Thermal Cycler (MJ Research, Poway, CA, USA) as follows: initial denaturation at 94 °C for 5 min, denaturation at 94 °C for 30 s followed by annealing at 60 °C, 45 s and extension at 72 °C, 1 min. This cycle was repeated 34 times followed by the final elongation step at 72 °C 5 min. PCR products were run in parallel with Fluo-DNA ladder (Zmtech) and detected by staining with 6X Fluo-DNA Loading Buffer (Zmtech).

### 2.2. Tissue Preparation

Time-pregnant dams were sacrificed at 14.5 dpc, embryos washed quickly in pre-chilled (4 °C) Phosphate Buffer Solution (PBS, pH7.4) and incubated overnight in 4% Paraformaldehyde/PBS at 4 °C (for paraffin embedding) or for 4 h (for cryopreservation). For paraffin preservation, embryos were washed in PBS, dehydrated in various concentrations of ethanol/PBS solution, and then embedded in paraffin (Histology Center, McGill University Health Center Research Institute); the paraffin-embedded blocks were stored at room temperature until use. For cryopreservation, the embryos were immersed in 15% sucrose/PBS at 4 °C for 4 h, then incubated in 30% sucrose/PBS at 4 °C on a rocking platform until the embryos sank to the bottom and were frozen in the histological molds filled with Tissue-Tek^®^ OCT compound (Sakura Finetek, Tokyo, Japan) by immersing the blocks into methylbutane bath on dry ice. The cryoblocks were stored at −80 °C until use.

### 2.3. Immunofluorescence

The following primary antibodies were used in this study: rabbit anti-Arl13b (1:150, Proteintech, Rosemont, IL, USA), rabbit anti-Calbindin D-28K (1:300, Calbiochem, Darmstadt, Germany), mouse anti-E-Cadherin (1:30, BD Transduction Laboratories^TM^, Franklin Lakes, NJ, USA), mouse anti-γ-tubulin (1:500, Sigma, St. Louis, MO, USA), mouse anti-PCNA (1:200, Abcam, Cambridge, MA, USA), rabbit anti-Six2 (1:80, Proteintech, USA), rabbit anti-WT1(C-19) (1:400, Santa Cruz, CA, USA), mouse anti-Neural cell adhesion molecule (1:200, Sigma Aldrich, Burlington, MA, USA). The following secondary antibodies were used: Alexa Fluor 546 donkey anti-mouse IgG (Invitrogen, Waltham, MA, USA), Alexa Fluor 488 donkey anti-mouse IgG (H + L) or Alexa Fluor 568 goat anti-rabbit IgG (H + L) (all from Invitrogen, USA), Alexa Fluor 647 goat anti-mouse IgG (H + L) (Jackson ImmunoResearch Laboratories Inc., West Grove, PA, USA).

For majority of the embryos, the blocks were processed to obtain sections of both kidneys. The sections with a visible central ureteric bud/collecting duct traversing the length of the tissue toward perimeter were considered “maximal” sections; 1-2 maximal sections per embryo were analyzed. A total of 4 µm E14.5 paraffin-embedded sections were deparaffinized and rehydrated in xylene and ethanol series using standard protocol. Antigen retrieval was performed in boiled Antigen Unmasking Solution (H-3300, Vector Labs, Burlingame, CA, USA) for 20 min. The sections were permeabilized with 1% Triton X-100/PBS for 1 h at room temperature and blocked with 3% Bovine Serum Albumin (BSA, Bioshop, Burlington, ON L7L 6A4, Canada), 10% normal goat/donkey serum (NGS/NDS, Jackson, ImmunoResearch Laboratories Inc., West Grove, PA, USA), 0.2% Triton X-100/PBS for 1 h at room temperature. Incubation with appropriate primary antibodies was performed at 4 °C overnight in 3% BSA, 3% NGS/NDS, 0.01% Triton X-100/PBS followed by washes in PBS, and incubation with the appropriate secondary antibody. Slides were washed in PBS and incubated in DAPI (4’, 6-Diamidino-2-Phenylindole, Dihydrochloride, Invitrogen, USA). To perform multiple staining, the sequential incubation with primary and secondary antibodies was done. For staining with Lotus Tetragonolobus Agglutinin (LTA-marker of proximal tubules, Vector Labs, USA) and Dolichos Biflorus Agglutinin (DBA -marker of collecting duct, Vector Labs, Burlingame, CA, USA), sections were incubated for 1 h at room temperature with LTA (1:200) or DBA (1:200) in 1% BSA, 0.01% Triton X-100 in PBS.

Cryopreserved 5 µm E14.5 sections were fixed for 10 min in 4% PFA/PBS at room temperature followed by antigen retrieval as described above. The sections were permeabilized with 1% Triton X-100/PBS for 30 min, washed in PBS, and blocked for 45 min in 3% BSA, 10% NDS, 0.1% Triton X-100. Sections were incubated with primary antibody overnight at 4 °C, then washed in 0.05% Tween 20/PBS and immunostained with appropriate secondary antibody. To perform the multicolor staining, the sections were incubated at room temperature in the mix of primary antibodies for 1 h 30 min, washed in 0.05% Tween-20/PBS and PBS and immunodetected with appropriate secondary antibodies diluted in 1%BSA, 1%NGS, 1%NDS/PBS in the presence of DAPI at RT for 1 h. Finally, sections were washed in 0.05% Tween 20/PBS and PBS. Slides were mounted with coverslips (ThermoFisher Scientific, Waltham, MA, USA) with a drop of ProLong^TM^ Gold Antifade Mountant (Invitrogen Molecular probe, Eugene, OR, USA). Images were acquired using AxioObserver 100 microscope via a Zeiss AxioCam MRm monochrome camera (Zeiss, Oberkochen, Germany). The Z-stack images of Arl13b & γ-tubulin&DBA &DAPI staining were taken using a confocal microscope (Zeiss, ELYRA Superresolution, Oberkochen Germany). The cilia were counted and measured using a magnification option only when cilia could be detected in the immediate proximity to the γ-tubulin-positive basal bodies.

### 2.4. Hematoxylin and Eosin Staining

E14.5 embryos of both genotypes were micro-dissected using Zeiss V8 stereomicroscope (Zeiss, Oberkochen, Germany); each kidney was paraffin embedded and serially sectioned at 4 µm. Hematoxylin and Eosin staining was serviced to the Histology Core Facility of the Research Institute of the McGill University Health Centre (RI-MUHC).

### 2.5. TUNEL Assay

The detection of apoptotic cells on E14.5 embryonic sections was carried out using the Terminal deoxynucleotidyl transferase (TdT) dUTP Nick-End Labeling (TUNEL) (Fluorescent-based Click-iT^®^ Plus TUNEL Assay, Invitrogen, Life Technologies, Carlsbad, CA, USA) according to the manufacturer’s instructions. Sections were further processed for Calbindin immunostaining and DAPI nuclear detection, and slides were mounted as described above.

### 2.6. In Situ Hybridization

The plasmids encoding various cDNAs were purified from *Escherichia coli* DH10β bacteria using standard protocols. The following cDNA probes were used: *c*-*Ret* [37] and *Gdnf* [38] (gift from Dr. Indra Gupta, McGill University), *Wnt11* [39] (gift from Dr. Paul Goodyer, McGill University), *Patched1* and *Gli1* (gift from Dr. Norman Rosenblum, University of Toronto). Plasmid cDNAs were linearized by digesting with appropriate endonuclease restriction enzymes; all restriction enzymes were either from New England Biolabs, Ipswich, MA, USA or Invitrogen Molecular probe, Eugene, OR, USA. The linearized cDNAs were extracted using a phenol/chloroform procedure or from the 0.8% low melt agarose gel using illustra^TM^ GFX^TM^ PCR DNA and Gel Band Purification Kit (GE Healthcare, Chalfont St Giles, UK). The linearized cDNA templates were digoxigenin (DIG)-labeled by in vitro transcription with an appropriate RNA polymerase according to the manufacture’s protocol (Roche^®^ Life Science Products, Oakville, ON, Canada) using T7 RNA polymerase (Invitrogen, Waltham, MA, USA), T3 RNA polymerase (Roche^®^ Life Science Products, Canada), Sp6 RNA polymerase (ThermoFisher Scientific, Waltham, MA, USA), DIG RNA labeling Mix (Roche,^®^ Life Science Products, Canada), RNAase –free DNase I, (ThermoFisher Scientific, USA), and RNase OUT^TM^ Recombinant Ribonuclease inhibitor (Invitrogen, USA). Antisense and sense riboprobes were synthesized in parallel. The DIG-labeled RNA probes were stored at −80 °C in aliquots until use.

Cryopreserved E14.5 embryos of both genotypes were sectioned at 10 µm, fixed in 4% PFA/PBS and treated with 10 µg/mL Proteinase K (Biotechnology, VWR, Radnor, PA, USA) for 10 min at 37 °C followed by the acetylation step. The acetylation solution (0.5% Acetic Anhydride in 0.1 M Triethanolamine) was freshly prepared and slides were incubated for 10 min and then washed in PBS. The slides were hybridized at 65 °C in the hybridization buffer (7.5 mM Tris-HCl, 1 mM Tris-base, 5 mM NaH_2_PO_4_·2H_2_O, 5 mM Na_2_HPO_4_, 5 mM EDTA, pH 8.0, 1x Denhardt’s solution, 50% deionized Formamide, 10% dextran sulfate, 1mg/ml yeast tRNA) with added DIG-labeled RNA probes (1:40,000 dilution) in a hybridization oven (Robbins Scientific^®^-1000, Sunnyvale, CA, USA) overnight in a sealed humidified chamber. The next day, the slides were washed twice for 30 min each at 65 °C in post-hybridization wash solution (1 X SSC (Saline Sodium Citrate), 50% Formamide, 0.1% Tween-20) followed by 2 washes in the maleic acid buffer with Tween (MABT: 0.15 M NaCl, 0.1 M Maleic acid, 0.1% Tween) for 30 min at room temperature, and in the RNA wash buffer (0.4 M NaCl, 10 mM Tris-HCl, pH 7.5, 5 mM EDTA) for 10 min at 37 °C. The slides then were washed 30 min at 37 °C in the RNA buffer supplemented with of 20 µM RNAse A (Invitrogen, Waltham, MA, USA) and blocked in 2% blocking reagent, 20% heat inactivated goat serum in the MABT for 2 h. Blocking solution was replaced by a solution of anti-DIG-Alkaline Phosphatase (AP) conjugate (Boehringer Mannheim, Biberach, Germany) diluted 1:1500 in blocking solution. The sections were incubated in the above solution overnight in humidified chamber at 4 °C. On the third day, the slides were washed four times, 15 min each, in the MABT then 2 times, at 10 min each, in the NTMT (100 mM NaCl, 100 mM Tris, pH 9.5, 50 mM MgCl_2_ 1%Tween 20) (2 mM levamisole were mixed in during the second NTMT wash). Finally, the slides were processed for coloration reaction with BM purple AP substrate (Roche^®^ Life Science Products, Oakville, ON, Canada) in the presence of 2 mM levamisole in a sealed humidified chamber in the dark, reaction was stopped in PBS wash once the precipitate formed. Slides were mounted in the ProLong^TM^ Gold Antifade Mountant (Invitrogen Molecular probe, Eugene, OR, USA). All solutions and plasticware were DEPC (Diethyl pyrocarbonate, Sigma-Aldrich, Burlington, MA, USA)-treated. All of the chemicals were from Sigma-Aldrige, USA or ThermoFisher Scientific, USA. The images were acquired on Aprio Turbo scanner using Imagescope software (both from Leica, Mannheim, Germany).

### 2.7. Analysis of Early Nephrogenesis

To study the nephrogenesis, the staining with the antibodies against NCAM (neural cell adhesion molecule, a specific marker for early nephrogenic structures [40]), and Calbindin D-28K was performed on the E14.5 kidney sections. The comparable sections of wildtype and *Fuzzy−/−* tissues were chosen for analysis. All of the NCAM-positive structures, representing early nephron structures, were morphologically stratified and counted into three groups representing the sequential development of nephrons, which are renal vesicle, comma-shaped body and S-shaped body. The total number of early nephron structures was counted in each kidney section. A total of 4 embryos per genotype and 2 kidneys per embryo were analyzed.

### 2.8. RNAseq and Bioinformatic Analysis

The total RNA was extracted using RNeasy Mini Kit (Qiagen, Germantown, MD, USA) using 3 kidneys from different embryos per genotype. RNA quantity was measure by NanoDrop™ Lite Spectrophotometer (ThermoFisher Scientific, Waltham, MA, USA), and RNA quality was analyzed by Bioanalyzer 2100 (Angilent, Santa Clara, CA, USA) with criteria of RIN number >7.0. The final cDNA libraries were prepared and sequenced on the Illumina NovaSeq platform through the massively parallel sequencing (MPS) service provided by Genome Quebec, Montreal, Canada. Stranded and Paired-end sequencing were performed. Raw reads were trimmed by “Trim Galore” https://github.com/FelixKrueger/TrimGalore for filtering out the low-quality bases and short reads. The trimmed reads were mapped to the mouse genome (Genome Reference Consortium Mouse Build 38-release 98) using HISAT2 [41]. After mapping, the HTSeq algorithm [42] was used to obtain read counts of genes based on the reference gene model (Mus_musculus.GRCm38.98.chr.gtf) obtained from Ensembl database. Differentially expressed genes were identified with statistical significance (*p* value < 0.05) by R package DeSeq2 [43]. To obtain insight into these differentially expressed genes, gene ontology analysis was conducted by using GOStats R package [44] for identifying enriched biological pathways. Python pandas module and Morpheus (https://software.broadinstitute.org/morpheus) were used for PCA analysis and heatmap data visualization.

### 2.9. Quantitative Polymerase Chain Reaction

E14.5 mouse kidneys were harvested and stored at −80 °C until use. RNA was extracted using RNeasy Mini Kit (Qiagen, Germantown, MD, USA), as described by the manufacturer. The reverse transcription reaction was performed using 1 μg of RNA with Superscript III polymerase (ThermoFisher Scientific, Waltham, MA, USA). The qPCR amplification was conducted using LightCycler^®^ 480 SYBR Green I Master mix (Roche^®^ Life Science Products, Oakville, ON, Canada) on a CFX384 quantitative PCR System (BioRad, Hercules, CA, USA) as recommended by the manufacturer. The following amplification parameters were used: 95 °C for 5 min, followed by 40 cycles of 5 s at 95 °C, 10 s at 65 °C and 10 s at 72 °C, followed by 5 s at 95 °C. Individual E14.5 kidneys were collected to extract RNA (biological replicates) and conduct RT-qPCR reactions. A total of 3 embryos per genotype, 1–2 kidneys from the same embryo per sample, were used. All of the experiments were conducted twice in triplicate for each sample. Primers are listed in Supplemental File S6.

### 2.10. Statistical Analysis

All data were collected in Microsoft Excel 2011 for Mac or PC and graphed using GraphPad Prism 6.0 software (La Jolla, San Diego, CA, USA). All data, unless specifically mentioned in the Figure legend, were expressed as mean values with error bars representing standard deviations (SD). An unpaired two tailed student t-test was used to determine difference between two groups. The multiple *t*-test was conducted for early nephrogenesis measurement. A fiducial limit of *p* ≤ 0.05 was used throughout. All statistical analyses were conducted using GraphPad Prism 6.0 software with exception for bioinformatics analysis.

## 3. Results

### 3.1. Morphological Analysis of Mutant Fuzzy−/− Kidneys

**Figure 1:**
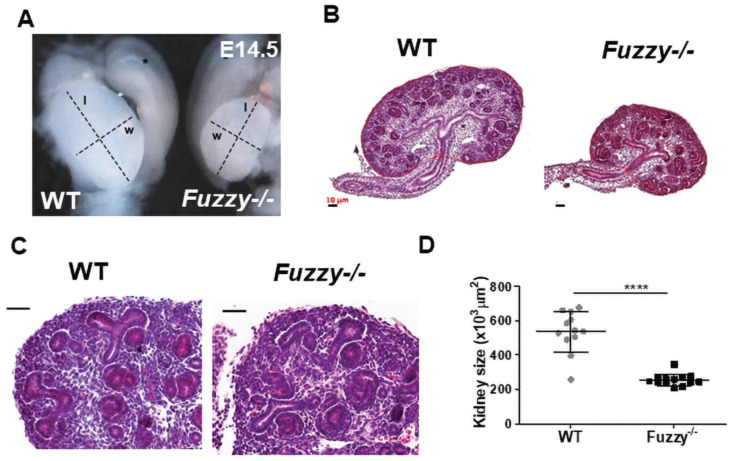
Renal hypoplasia in E14.5 *Fuzzy−/−* embryos. (**A**) Micro-dissected kidneys with attached gonads from wildtype and *Fuzzy−/−* E14.5 embryos; *w* = width, *l* = length, * gonads. (**B**) Maximal sections of paraffin-embedded and hematoxylin/eosin-stained wildtype and *Fuzzy−/−* kidneys; scale bars = 100 µm. (**C**) Morphological details of the wildtype and mutant kidneys; scale bars = 20 µm. (**D**) Statistical analysis of the maximal section size of wildtype and *Fuzzy−/−* kidneys. WT (total *n* = 11 sections), *Fuzzy−/−* (total *n* = 13 sections). 4 embryos per genotype, maximal sections from 2 kidneys per embryo were analyzed. **** *p* < 0.0001).

The majority of homozygous *Fuzzy−/−* mice in our colony do not survive past embryonic (E) day 14.5. Since the mammalian kidney starts to form around E10.5-11, we were able to harvest embryonic kidneys at E14.5. Analysis of the kidney in the mutant animals indicated a profound kidney hypoplasia in the *Fuzzy−/−* embryos (Figure 1); the size of heterozygous kidneys was indistinguishable from that of wildtype animals (not shown). Morphological evaluation of the hematoxylin/eosin-stained kidneys showed normally-appearing tubules and glomeruli (Figure 1B,C), yet mutant kidney size was significantly reduced (50–60%) compared to that of wildtype mice (Figure 1D).

**Figure 2:**
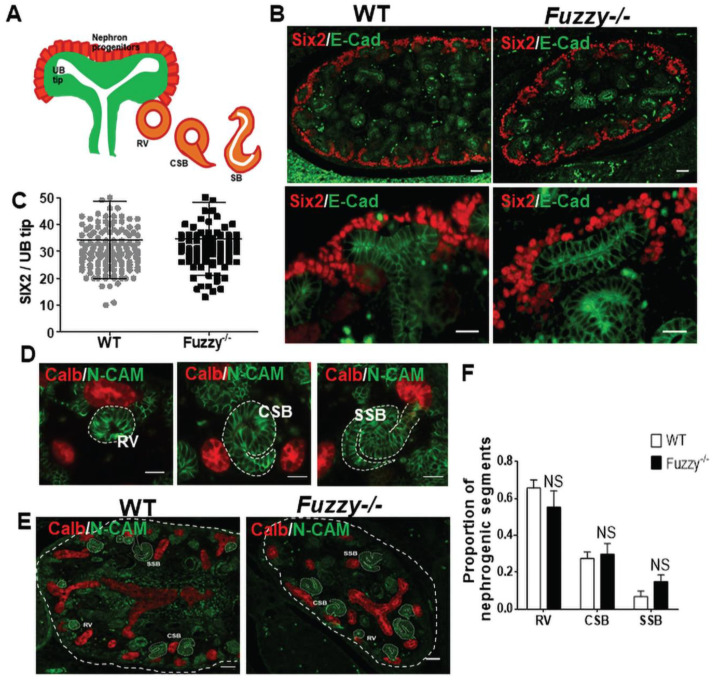
Early nephrogenesis in *Fuzzy−/−* embryonic kidneys. (**A**) Schemata of early nephrogenesis: nephron progenitor cells (red), UB tip (green), renal vesicle (RV), comma-shaped body (CSB), S-shaped body (SSB)–all depicted in orange. (**B**) Sections of wildtype and *Fuzzy−/−* embryos immunostained with anti-Six2 (red) and anti-E-Cadherin (green) antibodies; scale bars = 50 µM top panels and 25 µM bottom panels. (**C**) Statistical analysis of Six2+ cells per UB tip: WT (*n* = 166 UB tips) and *Fuzzy−/−* (*n* = 74 UB tips) from 4 embryos per genotype. (**D**) Morphological representation of RV, CSB and SSB identified by staining with anti-N-CAM antibody (green). Staining with anti-calbindin antibody (red) was used to visualize ureteric buds; scale bars = 25 µM. (**E**) Wildtype and *Fuzzy−/−* kidney sections immunostained as in D; scale bars = 50 µM. (**F**) Statistical analysis of early nephrogenesis: WT (total *n* = 101 developmental structures: RVs, CSBs & SSBs), *Fuzzy−/−* (total *n* = 103 developmental structures) from 4 embryos per genotype, 2 kidneys per embryo were analyzed. NS = not significant.

To ascertain which pathogenic mechanism(s) might be causing renal hypoplasia in *Fuzzy−/−* kidneys, we examined several developmental processes. First, we visualized the NPC pool, characterized by expression of Six2 [28,45]. The average number of the Six2+ cells around each ureteric bud (UB) tip was similar to that in wildtype animals (Figure 2A,C). High magnification imaging of the Six2+ cells did not reveal any changes in cell alignment around UB tip (Figure 2B). These results suggested that the initial specification and self-renewal of the NPCs in *Fuzzy−/−* kidneys was not affected.

As nephrogenesis proceeds, the NPCs move laterally around the UB tip, condense, and undergo mesenchyme-to-epithelial transition to form the earliest epithelialized nephron precursor with a central lumen, the renal vesicle (RV). RV epithelial cells express several specific transcription factors, ligands and adhesion molecules, including N-Cadherin (reviewed in [46]). The RV rapidly expands to form a comma-shaped body (CSB) and then an S-shaped body (SSB) (Figure 2D); the latter gives rise to all nephron tubular segments and the glomerulus. By counting N-cadherin-positive early nephron structures we found no differences in the RV:CSB:SSB proportions between mutant vs. wildtype E14.5 kidneys (Figure 2E,F), indicating that loss of Fuzzy function does not affect the progression through early stages of nephrogenesis.

**Figure 3:**
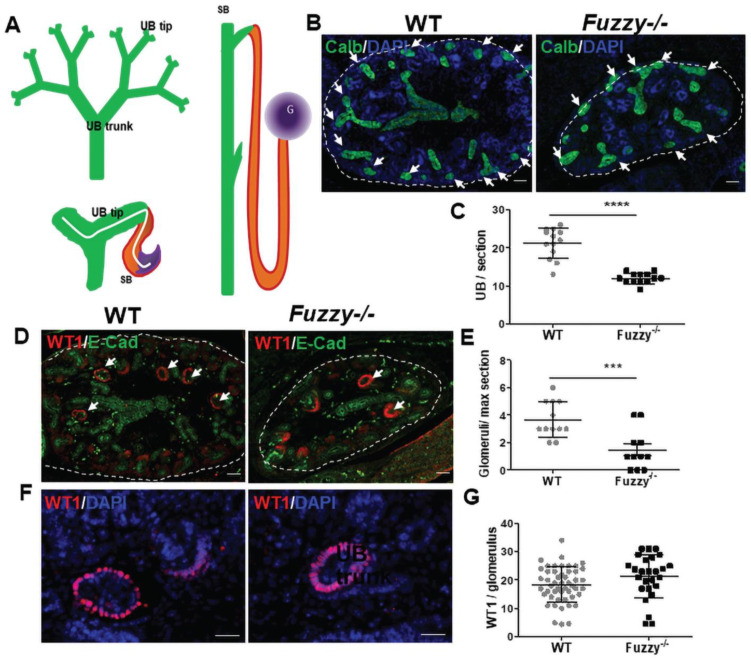
Defective ureteric branching morphogenesis in E14.5 *Fuzzy−/−* kidneys. (**A**) Schemata of UB branching and of a nephron. (**B**) Wildtype and *Fuzzy−/−* maximal sections immunostained with anti-Calbindin antibody (green) to visualize UBs and co-stained with DAPI (blue); arrows point at the UB tips, scale bars = 50 µM. (**C**) Statistical analysis of the number of UB tips per maximal section, WT (total *n* = 13 sections), *Fuzzy−/−* (total *n* = 13 sections) (**D**) Wildtype and *Fuzzy−/−* kidneys immunostained with anti-WT1 antibody (red) to detect glomerular podocytes and anti-E-Cadherin (green) to visualize UB branches; arrows point at glomeruli, scale bars = 50 µM. (**E**) Statistical analysis of the number of glomeruli per maximal section, WT (total *n* = 12 sections), *Fuzzy−/−* (total *n* = 11 sections). (**F**) Podocyte are visualized with anti-WT1 antibody (red) and co-stained with DAPI (blue); scale bars = 25 µM. (**G**) Statistical analysis of WT1+ podocyte per glomerulus, WT (total *n* = 44 glomeruli), *Fuzzy−/−* (*n* = total 20 glomeruli). For each measurement, maximal sections from 4 embryos per genotype, 2 kidneys per embryo were analyzed. *** *p* < 0.001, **** *p* < 0.0001.

The UB repeatedly branches to form a UB “tree” that ultimately defines both the number of nephrons and, thus, the initial kidney size. By visualizing UB tree with the anti-calbindin antibody in E14.5 kidney maximal cross-sections, we found that the number of UB branch tips significantly reduced in *Fuzzy−/−* mice (Figure 3B,C). The glomerulus is formed at the proximal aspect of the S-shaped body; the distal end of each S-shaped body is fused to its parent UB tip. Thus, the number of glomeruli reflects the extent of the UB branching, in the absence of abnormal NPC differentiation. Similar to the reduction in UB branch tip number, we detected fewer glomeruli per maximal cross-section in mutant kidneys (Figure 3D,E). A further analysis of podocytes did not show an abnormality in the mutant kidney: the number of podocytes per glomerulus was not reduced and their circumferential arrangement in the immature glomeruli was normal (Figure 3F,G). Thus, our results, indicate that renal hypoplasia in *Fuzzy−/−* kidneys is caused by defective ureteric branching morphogenesis.

### 3.2. Analysis of the Gdnf/c-Ret Signaling Axis in Fuzzy−/−Embryonic Kidneys

**Figure 4:**
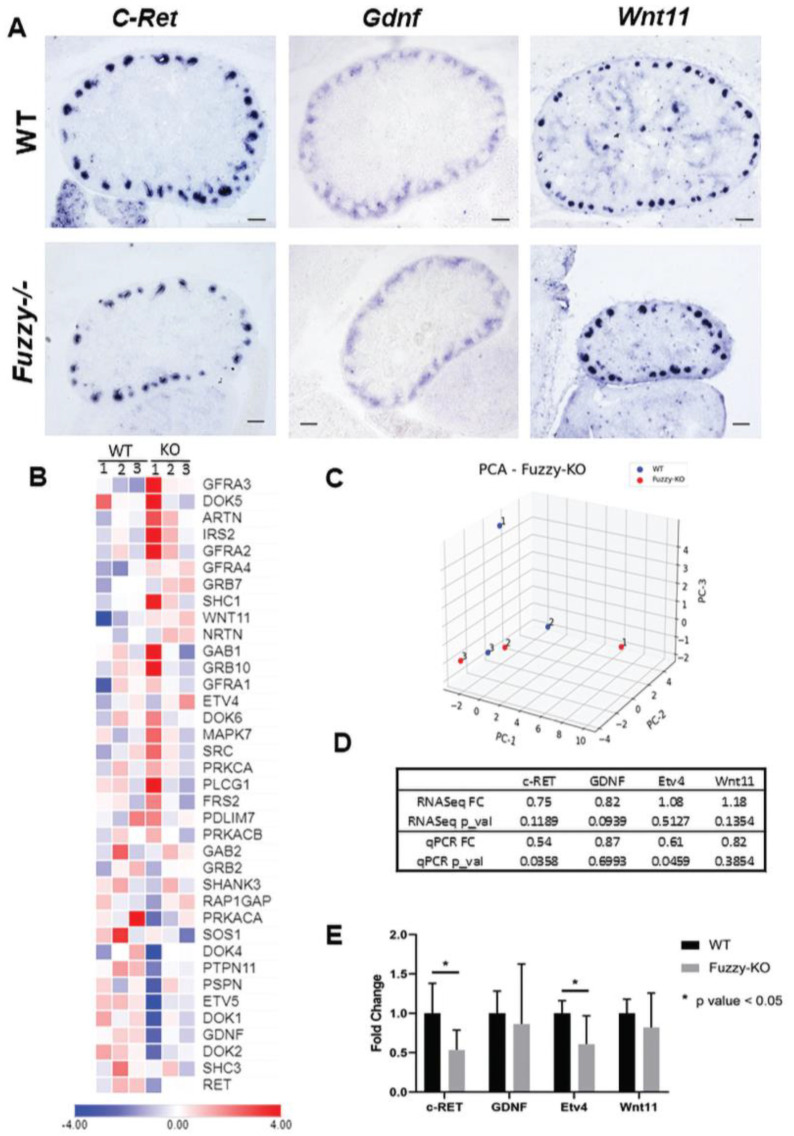
Gdnf/c-Ret signaling in E14.5. *Fuzzy−/−* embryonic kidneys. (**A**) In situ hybridization with antisense *c*-*Ret*, *Gdnf* and *Wnt11* cDNA probes on wildtype and *Fuzzy−/−* sections; scale bars = 100 µM; sections from 3 embryos per genotype were analyzed. (**B**) Heatmap of 39 Ret pathway genes from the RNAseq analysis of wildtype and *Fuzzy−/−* kidneys, 3 kidneys per genotype were used. (**C**) Principal component analysis of the Ret pathway genes; each sample per genotype is designated as 1, 2 or 3; this numeration corresponds to the order of samples in the heatmap. (**D**) Wildtype versus *Fuzzy−/−* fold change in the expression of selected Ret pathway genes analyzed by RNAseq and quantitative PCR. (**E**) Validation of selected Ret pathway genes by quantitative PCR. Standard errors of mean (SEM) are shown. 3 biological samples per genotype, 3 technical replicates per sample were used; the experiments were repeated twice. * *p* < 0.05.

The key signaling pathway that controls UB branching morphogenesis involves GDNF and its receptor, c-Ret (reviewed in [47]). Therefore, we analyzed expression of the genes in this pathway in mutant and wildtype kidneys. In situ hybridization to detect *c*-*Ret*, *Gdnf*, and *Wnt11* (a target of Gdnf/c-Ret signaling [39]) transcripts revealed no major differences (Figure 4A). We also used RNAseq analysis of E14.5 wildtype and mutant kidneys to interrogate expression of a broad panel of 39 c-Ret pathway genes (Figure 4B). For the majority of these genes, transcript levels were similar in mutant and wildtype kidneys; principal component analysis showed a lack of separation between mutant vs wildtype kidneys (Figure 4C). However, individual expression of *c*-*Ret*, *Gdnf*, and *Etv5* was mildly reduced, though the difference was not statistically significant (Figure 4B,C). By quantitative PCR, a small but significant reduction in the expression of *c*-*Ret* and *Etv4* in the mutant kidneys was detected (Figure 4E). Aligning with lower *c*-*Ret* expression, a small but significant reduction in cell proliferation in the cells of the UB tip ampullae, where GDNF/c-Ret signaling normally drives cell proliferation, was also noticed (Supplemental File S1). No differences in the apoptosis in the UB structures was noticed (Supplemental File S1).

### 3.3. Unbiased Analysis of Transcriptional Profiling in Fuzzy−/− vs. Wildtype Kidneys

**Figure 5:**
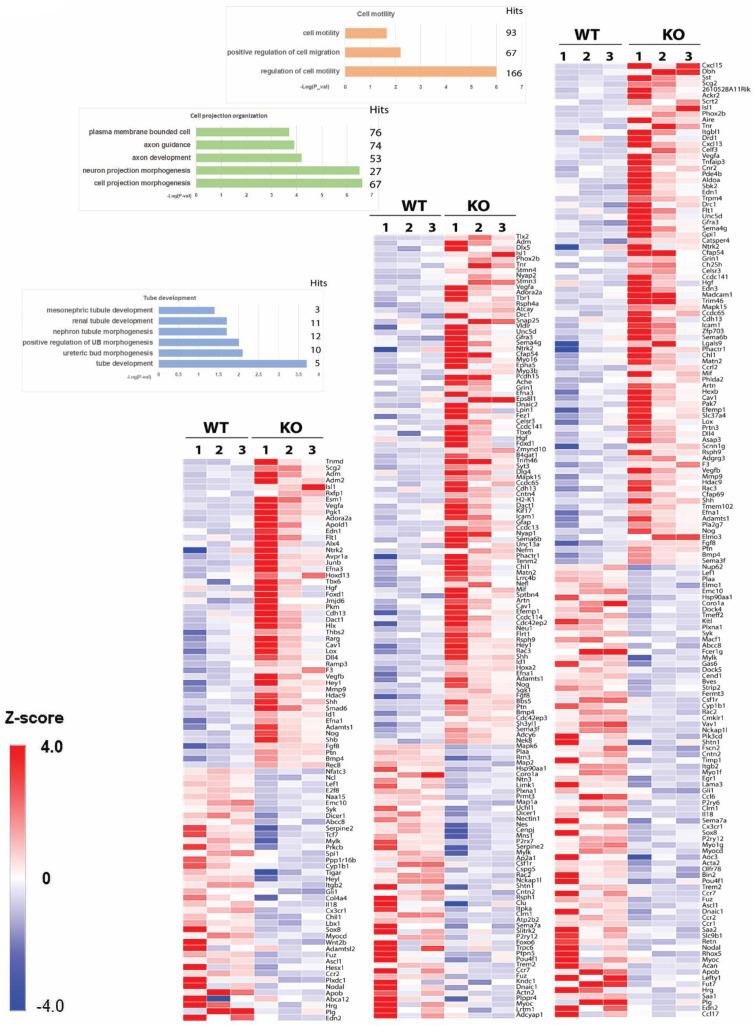
RNAseq analysis of *Fuzzy−/−* kidneys. Gene ontogeny (GO) pathway enrichment in wildtype and E14.5 *Fuzzy−/−* kidney RNA samples. The upregulated gene expression is depicted in red, downregulated gene expression is in blue. KO–*Fuzzy−/−* samples. Embryonic kidney(s) from each E14.5 embryo was used per sample; 3 embryos per genotype were dissected.

To identify Fuzzy-dependent molecules involved in kidney development and UB branching, we used an unbiased bulk-RNAseq approach. Embryonic kidney tissues from 3 embryos per genotype were used to construct cDNA libraries, and sequencing was performed in 2 batches. PCA analysis did not detect a significant batch effect (Supplemental File S2). In total, 1807 genes (1050 genes upregulated and 757 genes downregulated) were found differentially expressed in the *Fuzzy−/−* kidneys vs wildtype (*p* ≤ 0.05); a complete gene list is presented in Supplemental File S3. We then used GOStats R [44] to identify enhanced biological pathways and noted statistically significant gene enrichment for pathways regulating tube development (89 genes), cell projection organization (140 genes) and cell motility (159 genes) (Figure 5). The specific genes belonging to each GO pathway are listed in Supplemental File S4.

**Figure 6:**
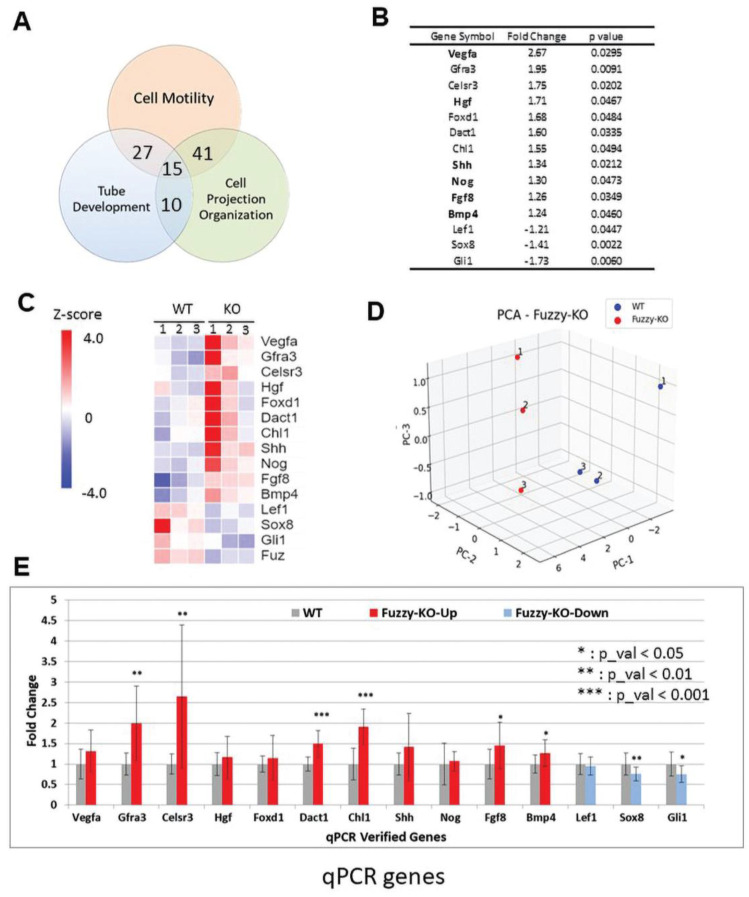
Specific genes with modified expression in *Fuzzy−/−* kidneys. (**A**) Venn diagram showing overlap between the genes in each two and all three GO groups. (**B**) List of genes chosen for further validation; in bold are the genes that overlap in 3 GO groups. (**C**) Heatmap of the selected genes; the last row is expression of *Fuzzy* mRNA; upregulated genes (UP) are depicted in red, downregulated (DOWN) genes are depicted in blue. (**D**) Principal component analysis for each of the 3 samples per genotype using 14 selected genes. Each sample per genotype is designated as 1, 2 or 3; the samples numeration in both PCA and heatmap is identical. Note a separation between the wildtype and *Fuzzy−/−* samples. (**E**) Validation of expression of 14 genes by quantitative PCR. Each biological sample was prepared from 1–2 kidneys from a E14.5 embryo, 4 embryos per genotype were used; the experiment was repeated twice. * *p* < 0.05; ** *p* < 0.01; *** *p*< 0.001.

Tubule formation relies on the complex cell behavior involving cell motility and formation of cell projections. Likewise, cell movements are powered by the ability to generate coordinated cell projections. Thus, we further enriched our selection for the genes common to 2/3 or 3/3 GO parameters. Out of 388 genes in the 3 GO groups, 78 genes were common for 2/3 GO parameters, and additional 15 genes were common for 3 GO pathways (Figure 6A); a detailed list of these overlapping genes is provided in Supplemental File S5. We selected 14 genes for further validation that include important growth and transcription factors with well-established roles in mammalian kidney development (Figure 6B,C). The expression of Fuzzy mRNA in the mutant kidneys was markedly reduced, as expected (Figure 6C, bottom row). PCA analysis demonstrated that the expression differences of the 14 selected genes were sufficient to separate the mutant and wildtype samples into 2 non-overlapping groups (Figure 6D). By quantitative PCR analysis, the expression of 8/14 genes were validated as being significantly different; the expression differences of the remaining 6/14 genes did not reach statistical significance but showed trends consistent with the RNAseq analysis (Figure 6).

### 3.4. Ciliogenesis and Shh Signaling in Fuzzy−/− Kidney

**Figure 7:**
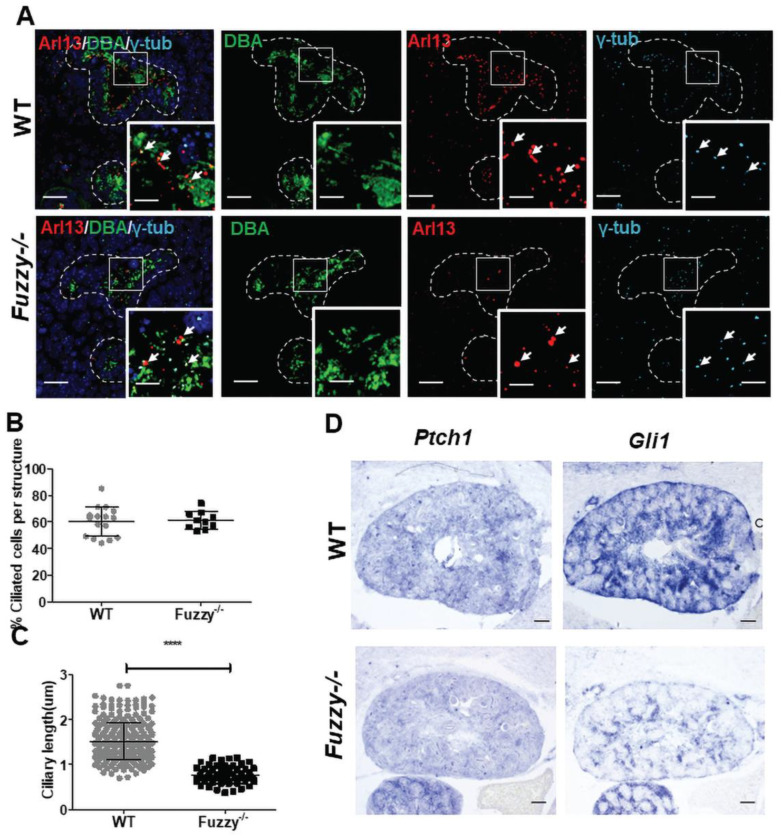
Ciliogenesis and Shh signaling in E14.5 *Fuzzy−/−* kidneys. (**A**) Confocal images of wildtype and *Fuzzy−/−* kidney sections immunostained with anti-Arl13b antibody (red) to detect primary cilium and anti-*γ*-tubulin antibody (light blue) to detect basal body; DBA (green) stains UB cells and DAPI (blue) stains nuclei. Intermittent white lines designate UB structures in which analyses of the ciliary length and numbers were carried out; scale bars = 25 µM. The inserts are the magnified areas within the UB tips designated by the squares. The cilia were counted only if both the Arl13b and γ-tubulin staining were detected for the same cilium; representative cilia/basal bodies are depicted by white arrows; insert scale bars = 8 µM. (**B**) Statistical analysis of ciliated cells in wildtype (*n* = 16 tissue fields) and *Fuzzy−/−* (*n* = 10 tissue fields). (**C**) Measurements of ciliary length in UB cells of wildtype (total *n* = 281 cilia) and *Fuzzy−/−* (total *n* = 67 cilia) were made using sections from 4 embryos per genotype. (**D**) In situ hybridization with antisense *Ptch1* (*Patched1*) and *Gli1* probes on wildtype and *Fuzzy−/−* sections; scale bars = 100 µM. 4 WT and 3 *Fuzzy−/−* embryos were analyzed. **** *p* < 0.0001.

Among the 14 selected genes, we noted *Shh* and its downstream effector *Gli1*. Shh signaling relies on the intact primary cilium [48]; we and others previously showed that loss of Fuzzy reduces ciliary length [21,22,24,49] and Shh signaling [20,21] in other tissues. We examined ciliary length in *Fuzzy* mutant UB cells and detected significantly shorter cilia, although the percentage of ciliated cells was similar in UB structures of both genotypes (Figure 7A–C). In situ hybridization for *Patched1* (Shh receptor) and *Gli1* transcripts was consistent with the RNAseq and qPCR results: there was no difference in *Ptch1* expression but there was a significant downregulation of *Gli1* in mutant kidneys (Figure 7D).

## 4. Discussion

**Figure 8:**
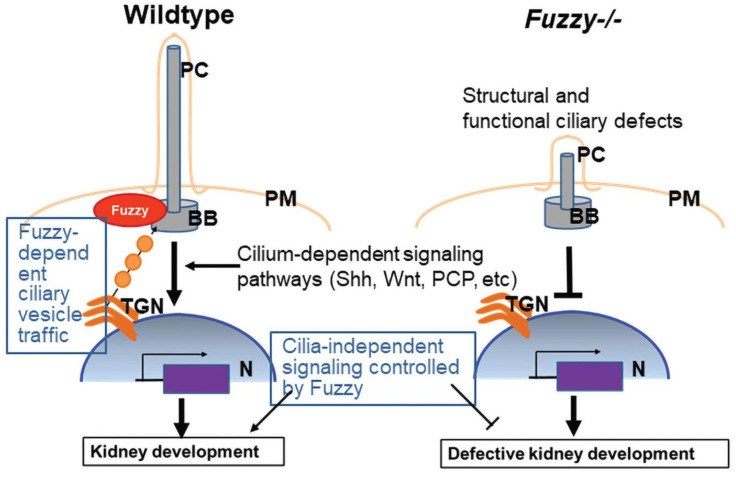
Model of Fuzzy involvement in kidney development. In the wildtype cells, Fuzzy-dependent vesicle trafficking that originates in Trans-Golgi Network (TGN) delivers cargo proteins necessary for structural and functional integrity of the primary cilium (PC) to the basal body (BB). Functional primary cilium controls multiple signaling pathways that may act via transcriptional regulation of downstream targets in the nucleus (N). Kidney development relies on timely localized expression of multiple transcription and growth factors; expression of some of these molecules is controlled by cilium-dependent signaling events. Potentially, Fuzzy could also control processes that do not rely on the primary cilium or transcriptional regulation (e.g., stabilization of cell projections necessary for directional cell movements). When Fuzzy is lost, all Fuzzy-dependent processes are deregulated, leading to defective kidney development.

Here, we report renal hypoplasia in a mouse with homozygous inactivating mutations of the PCP effector gene, *Fuzzy*, and attribute it to the disturbance of several molecular pathways during murine kidney development (Figure 8). Since a variety of mechanisms could have caused the renal hypoplasia, we systematically screened for abnormal specification, integrity of the nephron progenitor pool, defective UB branching morphogenesis, blocked mesenchyme-to-epithelial transition during early nephrogenesis, and dysregulated proliferation/apoptosis [46]. Morphological analysis of *Fuzzy−/−* embryonic kidneys revealed that UB branching morphogenesis was significantly affected, whereas the other developmental processes were relatively intact.

Interestingly, the Gdnf/c-Ret signaling axis [50] was mildly disturbed by the loss of *Fuzzy*. Other investigators have shown that null mutations of either *c*-*Ret* or *Gdnf* block initial UB outgrowth from the nephric duct, leading to renal agenesis in mice [51,52]; decreased activity of GDNF/c-Ret signaling leads to renal hypoplasia in both mice and humans [33,53]. Our RNAseq analysis of 39 Ret pathway gene expression found several significant differences between wildtype and *Fuzzy* mutant kidneys, including *c*-*Ret*, *Gdnf*, *Etv5*, or *Doc2*. By quantitative PCR, we confirmed that *c*-*Ret* expression significantly decreased in *Fuzzy−/−* kidneys.

Robust cell proliferation is required to generate an ampullae at the UB tip and drives the normal branching event; this is mediated by Gdnf/c-Ret signaling [54]. We detected a modest but significant decrease in the number of proliferating cells in UB ampullae. Since iterative branching leads to a logarithmic increase in nephron number during kidney development, a modest change in the UB tip cell proliferation and a modest delay in each branching event are predicted to have a substantial impact on final nephron number.

Unbiased RNAseq analysis pointed to the disturbance of the tubule formation through changes in cell movement and generation of cell projections. These events are plausible consequences of deficient PCP signaling. Indeed, we previously reported that *Fuzzy−/−* fibroblasts do not exhibit proper planar polarity, and this affects normal wound closure in vitro [24]. Our RNAseq analysis revealed significant changes in expression of several genes (e.g., *Vegfa*, *Bmp4*, *Shh*, *Fgf8*, *Lef1*, *Foxd1*, *Gli1*) that encode growth and transcription factors known to orchestrate kidney morphogenesis. We detected a significant increase in *Vegfa* expression in *Fuzzy−/−* mutant kidneys. Vascular endothelial growth factor (Vegfa) is expressed in maturing podocytes (where it guides development of the glomerular capillary [55]) and promotes UB branching as well [56,57]. Thus, the increased Vegfa expression could potentially be a compensatory response rather than a primary explanation for the branching defect. Morphological analysis of early glomeruli and podocytes in E14.5 *Fuzzy−/−* mutants showed no glomerular abnormalities, although early death of the *Fuzzy−/−* embryos before glomerular maturation precluded analysis of later glomerular stages.

We also detected a significant increase in *Bmp4* mRNA expression (both by RNAseq and qPCR). The timely reduction in Bmp4 by its modifier Gremlin1 in the mesenchyme surrounding each nephric duct is required for Gdnf/c-Ret dependent branching morphogenesis [58,59]; failure to suppress Bmp4 leads to renal agenesis [59]. Thus, our observations point at a potential role of Bmp4 dysregulation in renal hypoplasia of *Fuzzy−/−* mutants, although it is unclear how a disturbance of the PCP pathway alters *Bmp4* expression. Expression of fibroblast growth factor, *Fgf8*, was upregulated (both by RNAseq and qPCR). Although Fgf signaling is required for UB branching, aberrant FGF8 expression is common in ciliopathies and has been implicated in the craniofacial defects seen in *Fuzzy−/−* embryos [60,61].

In vertebrates, Fuzzy and other PCP effectors regulate ciliogenesis. The knockdown of PCP effectors in frogs or mutation of PCP effector genes in mice and humans cause “ciliopathy” phenotypes such as cyclopia (frogs) or polydactyly (mice, humans) [22,49,62]. Since the primary cilium serves as a signaling nexus in each cell, the disruption of the primary cilium may disturb intracellular signaling. For example, the Shh pathway relies on the integrity of the primary cilium [48]. In both *Xenopus* and mice, loss of *Fuzzy* was shown to affect Shh signaling by altering expression and activity of Shh activators Gli1 and Gli2 and the Shh inhibitor, Gli3 [20,21]. In the developing limbs of *Fuzzy−/−* and *Inturned−/−* mice, Gli3 processing is abnormal, affecting multiple Shh signaling target genes [20,23]. Similarly, the deregulation of Shh signaling during cranial development in *Fuzzy−/−* mice was recently reported [63]. Importantly, compromised Shh signaling leads to renal aplasia/dysplasia in both mice and humans through abnormal transcriptional regulation of Shh downstream targets that include *Wnt* and *c*-*Ret* genes [64]. In the present study, expression analysis of the Shh pathway genes by RNAseq and qPCR revealed changes in expression of *Gli1* and *Shh*, suggesting a potential deregulation of the downstream events in *Fuzzy−/−* kidneys.

In summary, we have demonstrated that null mutations in the PCP effector gene, *Fuzzy*, causes profound early renal hypoplasia in mice. Importantly, mutations in human homologs of the PCP effectors *INTURNED*, *FUZZY* and *WDPCP* have been associated with several human ciliopathies including Orofacial Digital Syndrome, Joubert Syndrome and Short-Rib-Polydactyly syndrome [49,62]. Renal hypoplasia was reported for some of these patients. Our work uncovers the pathogenic mechanisms underlying renal hypoplasia in mice with PCP effector mutations. We propose that PCP effector gene mutations likely cause congenital anomalies of the kidney and urinary tract (CAKUT) in some humans as well.

## Supplementary Materials

The following are available online at https://www.mdpi.com/article/10.3390/jdb10010001/s1, File S1: Proliferation and apoptosis in E14.5 *Fuzzy−/−* UB tip cells, File S2: Batch effect analysis of the gene expression by RNAseq, File S3: RNAseq list of upregulated and downregulated genes with significant changes in gene expression in E14.5 *Fuzzy−/−* kidneys comparing to control tissues, File S4: RNAseq gene list grouped by Gene Ontogeny parameters, File S5: List of genes that overlap in the three chosen GO parameters, File S6: List of primers used for quantitative PCR.
