# Supplementary material for: Loss of Planar Cell Polarity Effector Fuzzy Causes Renal Hypoplasia by Disrupting Several Signaling Pathways
Source: J Dev Biol. 2021 Dec 23;10(1):1. doi: 10.3390/jdb10010001 (PMC8788523; doi:10.3390/jdb10010001)
Supplement: Supplementary file 1 [file jdb-10-00001-s001.zip › Supplementary File S1.pdf]

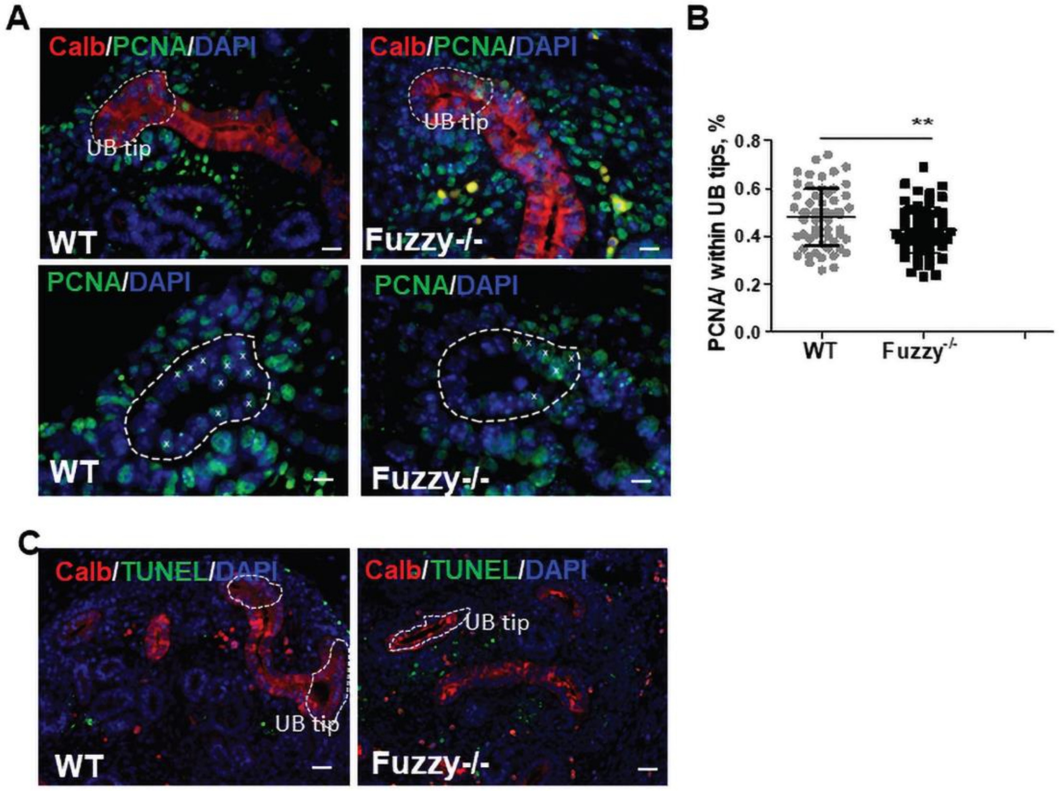

Supplementary File S1. Proliferation and apoptosis in E14.5 Fuzzy−/− UB tip cells. (**A**) Wildtype and *Fuzzy*−/− kidney sections were immunostained with anti-Calbindin (red) antibody to visualize UB structures, anti-PCNA antibody (green) to identified proliferating cells and co-stained with DAPI (blue). Since the cells within UB tips are specifically characterized by high proliferation level, the number of the PCNA+ cells was counted within UB tips (UB ampullae) designated by the intermittent white lines. Each cell counted is marked with a small white cross; *scale bars* –20 and 10μM at the top and bottom images, respectively. (**B**) Statistical analysis of PCNA+ cells in the wildtype UB tips (total *n* = 67 structures) and *Fuzzy*−/− (*n* = total 60 structures) from 4 embryos per genotype, 2 kidneys per embryo. (**C**) Wildtype and *Fuzzy*-/- kidney sections were immunostained with anti-Calbindin antibody (red) to identify UB structures; apoptotic cells (green) are TUNEL reagent positive, DAPI (blue) stains nuclei. Apoptotic cells were counted within UB tips designated by the intermittent white lines. Presence of very rare apoptotic cells within UB tips precluded statistical analysis. Sections from 4 embryos per genotype, 1–2 kidneys per embryo were analyzed.
