# Supplementary material for: Loss of Planar Cell Polarity Effector Fuzzy Causes Renal Hypoplasia by Disrupting Several Signaling Pathways
Source: J Dev Biol. 2021 Dec 23;10(1):1. doi: 10.3390/jdb10010001 (PMC8788523; doi:10.3390/jdb10010001)
Supplement: Supplementary file 1 [file jdb-10-00001-s001.zip › Supplementary File S2.pdf]

Supplemental File 2. Batch Effect Analysis

Principal Component Analysis for Batch effect

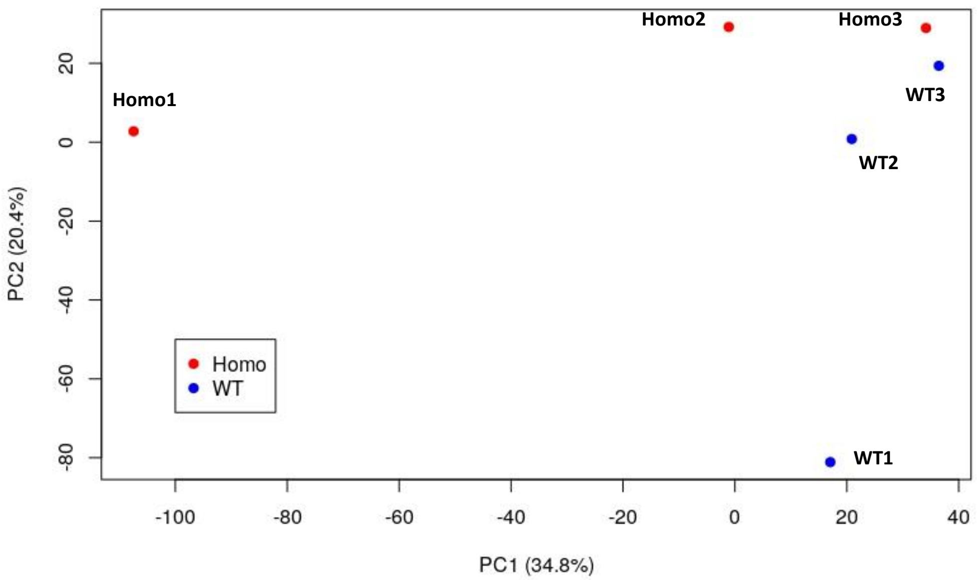

Sample	Batch
WT1	1
HOMO1	1
WT2	1
HOMO2	1
WT3	2
HOMO3	2

1. There are 2 batches of runs for interlaced 6 samples.
2. The PCA shows that there is **no** batch effect.
