# Supplementary material for: Loss of Planar Cell Polarity Effector Fuzzy Causes Renal Hypoplasia by Disrupting Several Signaling Pathways
Source: J Dev Biol. 2021 Dec 23;10(1):1. doi: 10.3390/jdb10010001 (PMC8788523; doi:10.3390/jdb10010001)
Supplement: Supplementary file 1 [file jdb-10-00001-s001.zip › Supplementary File S5.pdf]

Supplemental File 5. Genes hits in GO analysis

Cell Motility & Tube Development Total:42	FC	p_val
**Vegfa**	2.67	0.0295
**Hgf**	1.71	0.0467
**Shh**	1.34	0.0212
**Fgf8**	1.26	0.0349
**Nog**	1.30	0.0473
**Bmp4**	1.24	0.0460
Isl1	4.72	6.9E-06
Ntrk2	1.91	0.0286
Cdh13	1.63	0.0455
Cav1	1.48	0.0141
Efna1	1.31	0.0350
Adamts1	1.31	0.0362
Ptn	1.25	0.0095
Mylk	0.72	0.0273
Fuz	0.41	1.9E-06
**Lef1**	0.83	0.0447
**Gli1**	0.58	0.0060
**Sox8**	0.50	0.0022
Scg2	5.71	3.5E-05
Edn1	2.13	0.0041
Flt1	2.02	0.0109
Lox	1.47	0.0380
Dll4	1.46	0.0076
F3	1.37	0.0093
Vegfb	1.36	0.0314
Mmp9	1.35	0.0310
Hdac9	1.35	0.0318
Emc10	0.80	0.0144
Syk	0.77	0.0163
Abcc8	0.74	0.0497
Cyp1b1	0.68	0.0038
Itgb2	0.59	0.0034
Il18	0.52	0.0141
Cx3cr1	0.51	0.0002
Myocd	0.48	4.0E-05
Ascl1	0.37	5.2E-05
Ccr2	0.35	1.4E-06
Nodal	0.27	0.0110
Apob	0.19	1.1E-04
Hrg	0.09	0.0070
Plg	0.05	0.0016
Edn2	0.05	0.0013

Cell Motility & Cell Projection Organization Total: 56	FC	p_val
**Vegfa**	2.67	0.0295
**Hgf**	1.71	0.0467
**Shh**	1.34	0.0212
**Fgf8**	1.26	0.0349
**Nog**	1.30	0.0473
**Bmp4**	1.24	0.0460
Isl1	4.72	6.9E-06
Ntrk2	1.91	0.0286
Cdh13	1.63	0.0455
Cav1	1.48	0.0141
Efna1	1.31	0.0350
Adamts1	1.31	0.0362
Ptn	1.25	0.0095
Mylk	0.72	0.0273
Fuz	0.41	1.9E-06
**Gfra3**	1.95	0.0091
**Celsr3**	1.75	0.0202
**Chl1**	1.55	0.0494
Phox2b	4.57	0.0012
Tnr	4.33	0.0068
Drc1	2.08	0.0361
Unc5d	1.98	0.0137
Sema4g	1.94	0.0052
Cfap54	1.89	0.0080
Grin1	1.81	0.0477
Ccdc141	1.73	0.0175
Trim46	1.65	0.0164
Mapk15	1.63	2.2E-05
Ccdc65	1.63	0.0293
Icam1	1.59	0.0114
Sema6b	1.56	0.0390
Phactr1	1.55	0.0211
Matn2	1.54	0.0261
Mif	1.51	0.0168
Artn	1.49	0.0323
Efemp1	1.48	0.0317
Rsph9	1.40	0.0189
Rac3	1.35	0.0310
Sema3f	1.22	0.0442
Plaa	0.82	0.0250
Hsp90aa1	0.79	0.0423
Coro1a	0.79	0.0457
Plxna1	0.78	0.0123
Csf1r	0.68	0.0260
Rac2	0.67	0.0043
Nckap1l	0.64	4.6E-04
Shtn1	0.63	0.0387
Cntn2	0.61	0.0255
Clrn1	0.52	1.2E-06
Sema7a	0.51	0.0191
P2ry12	0.50	0.0111
Pou4f1	0.44	0.0301
Trem2	0.44	5.6E-04
Ccr7	0.44	0.0426
Dnaic1	0.35	0.0099
Myoc	0.20	0.0190

Tube Development & Cell Projection Organization Total: 25	FC	p_val
**Vegfa**	2.67	0.0295
**Hgf**	1.71	0.0467
**Shh**	1.34	0.0212
**Fgf8**	1.26	0.0349
**Nog**	1.30	0.0473
**Bmp4**	1.24	0.0460
Isl1	4.72	6.9E-06
Ntrk2	1.91	0.0286
Cdh13	1.63	0.0455
Cav1	1.48	0.0141
Efna1	1.31	0.0350
Adamts1	1.31	0.0362
Ptn	1.25	0.0095
Mylk	0.72	0.0273
Fuz	0.41	1.9E-06
**Foxd1**	1.68	0.0484
**Dact1**	1.60	0.0335
Adm	5.65	0.0109
Adora2a	2.31	0.0012
Efna3	1.81	0.0218
Tbx6	1.72	0.0488
Hey1	1.36	0.0414
Id1	1.33	0.0030
Dicer1	0.76	0.0155
Serpine2	0.73	0.0316
